# Best practices in DNA methylation: lessons from inflammatory bowel disease, psoriasis and ankylosing spondylitis

**DOI:** 10.1186/s13075-019-1922-y

**Published:** 2019-06-03

**Authors:** Jessica M. Whyte, Jonathan J. Ellis, Matthew A. Brown, Tony J. Kenna

**Affiliations:** 1 Institute of Health and Biomedical Innovation, Queensland University of Technology, Woolloongabba, Queensland Australia; 2 Translational Research Institute, Princess Alexandra Hospital, 37 Kent Street, Woolloongabba, Queensland 4102 Australia

**Keywords:** DNA methylation, Epigenetics, Ankylosing spondylitis, Psoriasis, Inflammatory bowel disease (IBD), Human

## Abstract

Advances in genomic technology have enabled a greater understanding of the genetics of common immune-mediated diseases such as ankylosing spondylitis (AS), inflammatory bowel disease (IBD) and psoriasis. The substantial overlap in genetically identified pathogenic pathways has been demonstrated between these diseases. However, to date, gene discovery approaches have only mapped a minority of the heritability of these common diseases, and most disease-associated variants have been found to be non-coding, suggesting mechanisms of disease-association through transcriptional regulatory effects.

Epigenetics is a major interface between genetic and environmental modifiers of disease and strongly influence transcription. DNA methylation is a well-characterised epigenetic mechanism, and a highly stable epigenetic marker, that is implicated in disease pathogenesis. DNA methylation is an under-investigated area in immune-mediated diseases, and many studies in the field are affected by experimental design limitations, related to study design, technical limitations of the methylation typing methods employed, and statistical issues. This has resulted in both sparsity of investigations into disease-related changes in DNA methylation, a paucity of robust findings, and difficulties comparing studies in the same disease.

In this review, we cover the basics of DNA methylation establishment and control, and the methods used to examine it. We examine the current state of DNA methylation studies in AS, IBD and psoriasis; the limitations of previous studies; and the best practices for DNA methylation studies. The purpose of this review is to assist with proper experimental design and consistency of approach in future studies to enable a better understanding of the functional role of DNA methylation in immune-mediated disease.

## Background

Ankylosing spondylitis (AS), inflammatory bowel disease (IBD) and psoriasis are characterised by systemic or organ-specific failures of the regulatory pathways of the immune system resulting in uncontrolled inflammation. These ‘seronegative’ diseases are closely related clinically and often co-occur in patients and families. Several GWAS in these diseases have identified the same genes that confer susceptibility to disease, including human leukocyte antigen (*HLA*), which plays a vital role in adaptive immunity and tolerance, IL-23 receptor (*IL23R*), DNA methyltransferase 3A (*DNMT3A*), *DNMT3B*, *DNMT3L* and several genes involved in the JAK-STAT pathway [1–4]. A cross-disease genetic study of five seronegative diseases (AS, ulcerative colitis, Crohn’s disease, psoriasis and primary sclerosing cholangitis) identified pleiotropic genes and shared pathogenic pathways between these diseases [4].

Despite the numerous disease-associated variants identified in AS, IBD and psoriasis, they cumulatively explain only a small proportion (< 28%) of the heritability of these diseases [4]. Potential reasons for this ‘missing heritability’ include large numbers of variants of smaller effect yet to be identified, rare variants being missed by available genotyping arrays, copy number variation (CNV), insertion/deletion events, gene-gene interactions and epigenetic factors. Epigenetics refers to functional modifications to DNA other than base sequence coding and includes histone modifications, non-coding RNA interactions with transcriptional and translational machinery and DNA methylation. Epigenetic variation is a dynamic and responsive process that occurs throughout life and in each individual cell and tissue within a single organism.

In this review, we focus on DNA methylation as it has the most robust measurement methods of all forms of epigenetic variation, making it a tractable epigenetic form to study. We discuss the current state of epigenetic research in AS, IBD and psoriasis, and the key aspects of study design that are relevant to dissecting the pathogenic mechanisms involved in these diseases.

## What is DNA methylation?

DNA methylation refers to the addition of a methyl group (CH_3_) to a cytosine to form 5-methylcytosine (5mC). Predominantly, methylation occurs on cytosine phosphate guanine-paired bases (CpGs). The majority of methylated CpGs occur in CpG islands, dense CpG regions of DNA between 300 and 3000 bp. CpG sites located 2 kb upstream or downstream of a CpG island are defined as CpG shores, and CpG sites 2 kb beyond these shores are defined as CpG shelves. Regions outside this 4 kb stretch are referred to as the ‘open sea’. DNA methylation can occur in cytosines outside CpG sites, but whether these are recognised as methylated sites is unclear [5]. Generally, CpGs in differentiated cells are uniformly methylated or unmethylated between homologous chromosomes and within cell populations, resulting in a bimodal distribution.

DNA methylation is a highly stable chemical marker that is maintained through mitosis. It is moderately heritable between generations. Maternal exposure to environmental factors can affect embryos in utero and, for female embryos, also affect their gametes, as female reproductive cells are fixed at birth [6]. As cells undergo two cycles of demethylation during reproduction, it is unclear whether these changes can be transmitted beyond these generations [6, 7].

**Fig. 1:**
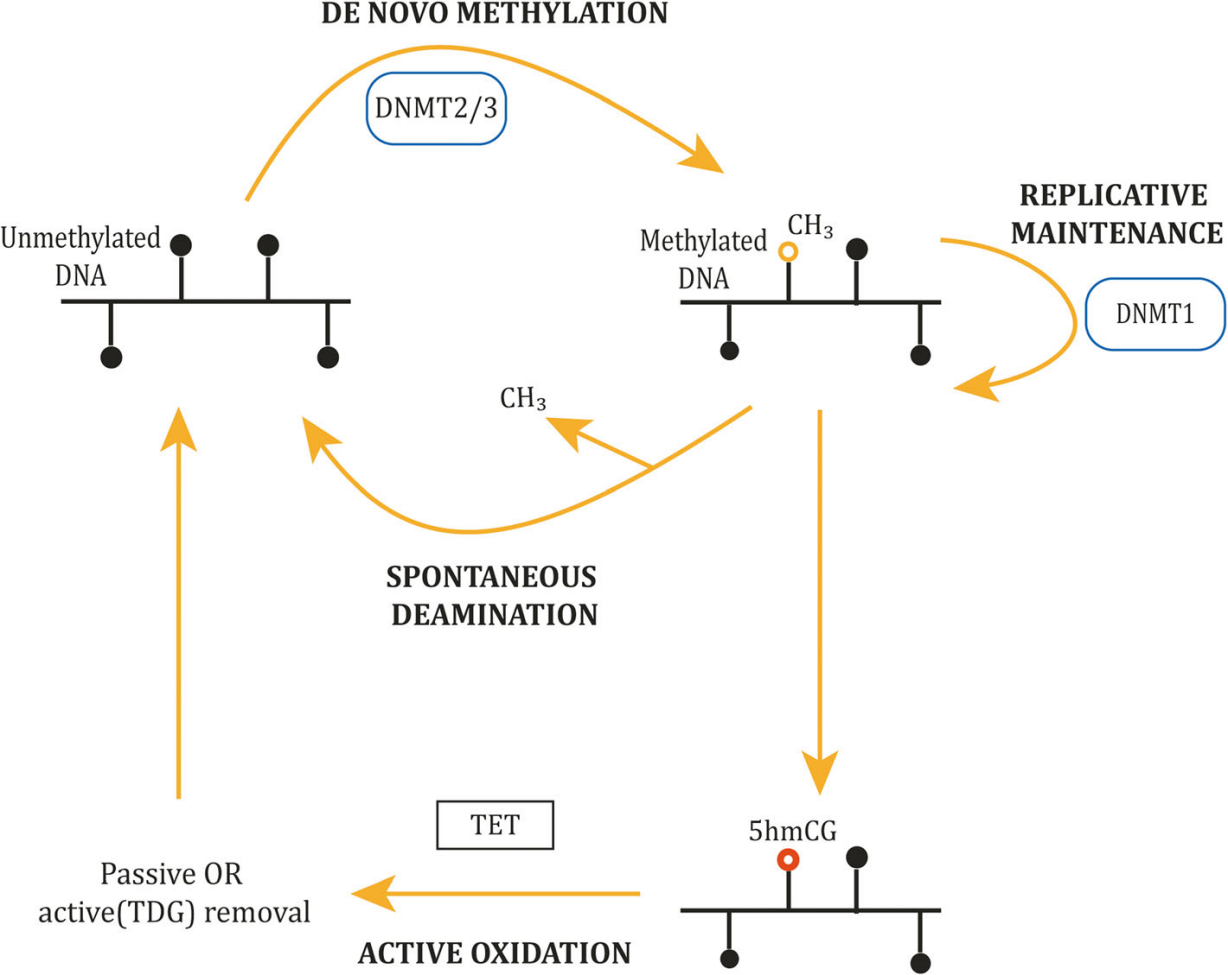
The process of DNA methylation addition, maintenance and removal. The de novo establishment of DNA methylation is carried out by DNMT2, DNMT3A, DNMT3B and DNMT3L. Once established, DNA methylation requires maintenance to prevent loss of methylation either spontaneously through ‘passive’/spontaneous deamination or actively by the ten-eleven translocation (TET) enzymes. The TET protein family directly remove DNA methylation markers through successive oxidation steps followed by removal of thymine DNA glycosylase (TDG)

The process of methylation establishment and maintenance is carried out by the DNA methyltransferase (DNMT) protein family (see Fig. 1). DNMT family proteins have a CXXC domain that recognises unmethylated CpGs [8]. The de novo establishment of DNA methylation is carried out by DNMT2, DNMT3A, DNMT3B and DNMT3-like (DNMT3L). Once established, DNA methylation requires maintenance to prevent loss of methylation either spontaneously through ‘passive’ deamination or through active deamination by the recently discovered ten-eleven translocation (TET) enzymes. Deamination is the spontaneous loss of an amino group. Methylated cytosines deaminate to thymine, a stable alternative nucleotide, resulting in the gradual depletion of cytosine in the genome. Cytosine to thymine mutations are the most common mutations observed in mammals. The asymmetrically methylated paired bases resulting from deamination are preferentially recognised and methylated by DNMT1, which also maintains DNA methylation during cellular replication.

Initially considered a ‘switch’ for gene activation or silencing, the exact effect of DNA methylation on gene expression is highly context-dependent, with promoter DNA methylation associated with gene silencing, whereas DNA methylation in the effector region is associated with gene activation. DNA methylation has been investigated in disease for many purposes as follows: as a potential biomarker for disease outcome/severity, to elucidate transcriptional control and to determine the effect of genetic changes on function. Local DNA sequence is the primary determinant of DNA methylation state [9]. Disease-associated SNPs can therefore alter DNA methylation patterns to affect gene expression and cellular function, either in *cis* (at the gene itself), or *trans* (indirectly, often distantly).

## Factors influencing DNA methylation

Analysis of DNA methylation in disease greatly benefits from an experimental design that considers the intrinsic and extrinsic factors that affect DNA methylation. We highlight below some of the well-established and identifiable influencers of DNA methylation.

### Age

Embryonic development is a crucial period for the establishment of DNA methylation patterns, and prenatal exposure to stressful environmental factors can alter DNA methylation patterns in adulthood (some sex-specific) [10, 11]. Subsequent age-related changes are observed in all cell and tissue types. These changes are indicative of biological ageing to a greater extent than chronological ageing and are related to both cellular intrinsic factors as well as changes in cell composition in whole blood [12, 13].

### Sex

Whilst differences in DNA methylation of sex chromosomes and sex-related genes are well characterised, identifying differences in DNA methylation of other autosomal sites between sexes has been inconsistent [14]. Further research is needed to confirm these findings and to determine if these differences persist to adulthood.

### Smoking

Smoking markedly influences DNA methylation. Smoking-related changes in DNA methylation are correlated with smoking severity, whilst some CpG sites revert to never smoking DNA methylation levels within 12 weeks after smoking cessation and others persist after 10 years [15]. These changes are cell type-specific [16]. Second-hand smoke exposure affects DNA methylation similarly to direct exposure [17]. Emerging data indicates e-cigarettes, or vaping, also alter epigenetic and transcriptional regulation [18].

### Medication

Many medications used in the treatment of inflammatory diseases influence DNA methylation, including prescribed and ‘complementary’ medications. For example, glucocorticoids, non-steroidal anti-inflammatory drugs (NSAIDs), sulfasalazine, methotrexate and tumour necrosis factor-α inhibitors all alter DNA methylation [19, 20].

### Alcohol and diet

Alcohol is known to alter DNA methylation even at low levels (1 drink/week); however, the persistence and breadth of its effect is poorly understood [21]. Dietary factors can also affect DNA methylation, although this has predominantly been examined in obesity or cancer intervention studies. LINE (long interspersed nuclear element), a surrogate for global DNA methylation levels, has been indicated as a general diet response marker [22]. A single study was able to link levels of LINE methylation to serum glucose levels, and the level of hydroxymethylation to BMI, waist circumference and total cholesterol [23]. BMI can alter DNA methylation levels as a consequence of adiposity [24]. Adiposity is associated with increased inflammation markers and can predispose to inflammatory diseases [25].

### Sample cell type

Cell types each have unique DNA methylation profiles, and differences in tissue DNA methylation patterns are strongly influenced by the specific mixtures of cell types within tissues [26]. Further, the effect of environmental factors and disease on DNA methylation patterns is cell type-specific. In silico deconvolution methods are available for estimating cell proportions, and many of these methods have been described in a recent review which also provides insight into when different methods should be applied [27].However, these methods do not enable the identification of cell-type specific changes in DNA methylation nor control for the dilution of cell specific signals within complex samples.

## Measuring DNA methylation

The gold standard method for characterising DNA methylation is bisulfite conversion, which converts unmethylated (but not methylated) cytosine to uracil enabling the identification of methylated regions through sequencing. An additional oxidative reaction can be used to identify hydroxymethylated regions (5hmC) but is rarely used due to additional cost and input requirements. DNA methylation measurement techniques can be broadly categorised as bisulfite based, DNA methylation-sensitive restriction enzyme based, methylcytosine-specific antibody methods, enrichment methods or direct sequencing (see Table 1). Bisulfite methods are favoured for genome-wide studies; however, as most of the genome is incapable of being methylated, this is often paired with enrichment or selection methods.

**Table 1:** Summary table of methods for the detection of DNA methylation

Approach	Method	Relative cost	Throughput	Resolution	Advantages	Disadvantages
Bisulfite-based methods	Methylation-specific PCR (MSP)	+	+	Region	• Cheap • Easy • Relatively quick	• Single-gene resolution • No high-throughput capability • Amplification-based
	Human MethylationEPIC BeadChip array	++	+++	Base pair/region	• High throughput • Targeted to functionally important regions	• Probe variation • Selected regions (biased)
	Single-cell nucleosome methylation transcription sequencing (scNMTseq)	++++	++++	Base pair	• Single-cell resolution • Low-input numbers • Nucleosome, epigenetic and transcription from a single cell	• Expensive • Analysis methods complex • Amplification required
	Reduced representation bisulfite sequencing (RRBS)	++	++	Base pair	• Covers CpG dense regions	• Specific base sequence selection due to enzymatic cut sites • Cannot distinguish 5mC from 5hmC
	Pyrosequencing	+++	++++	Base pair	• Quantitative • High throughput	• Expensive • Non-targeted
	Whole genome bisulfite sequencing (WGBS)	++++	+++++	Base pair	• Genome-wide coverage • Sequence/SNP information • A large amount of data	• Expensive • A large amount of data • Large areas that are incapable of being methylated
Methyl-CpG isolation-based methods	Methylated DNA immunoprecipitation (MeDIP)	++	++	Region	• Can incorporate with PCR/microarray/NGS • 5mC in dense, less dense and repeat regions are covered • Antibody-based selection is independent of sequence	• Lower base-pair resolution (~ 150 bp) • Potential antibody non-specific interactions • Antibody-based selection biased towards hypermethylated regions • Unmethylated regions can only be interpreted from the absence of signal
	methyl-CpG-binding domain-isolated genome sequencing (MIGS)	++	++	Region	• Genome-wide 5mC coverage	• Lower base-pair resolution (~ 150 bp) • No 5hmC coverage • Bias towards hypermethylated regions
	Combined bisulfite conversion and restriction analysis (COBRA)	++	+	Base pair/region	• Small input • Targeted approach	• No high-throughput capability
	HpaII tiny fragment enrichment by ligation-mediated PCR (HELP-tagging assay)	++	+	Base pair	• Enrichment of areas of interest • Easy	• HpaII only recognises CCGG when the middle cytosine is unmethylated
	Methylation-specific amplification microarray (MSAM)	++	++	Base pair	• Broad coverage • Customizable	• Amplification-based • Biased to regions selected • Non-direct measure
Kinetics-based methods	PacBio single-molecule, real-time (SMRT) sequencing	+++	++++	Base pair	• Long reads • Bisulfite conversion free	• Expensive • Covers
	Mass spectrometry	++++	+	Base pair	• Direct • Amplification-free method	• Expensive • Input requirement high without additional targeted selection
Optical biosensing	Fluorescence resonance emission transfer (FRET)	++++	++	Base pair	• Amplification-free method • Capable of miniaturisation	• Expensive • Not user-friendly data analysis packages
	Surface plasmon resonance (SPR)	++++	+++	Base pair	• Amplification-free method • Bisulfite conversion free • Low-sample input	• Expensive • Difficult to analyse • Complex to run
Electrochemical biosensing	Graphene or gold affinity methods	+++	+++	Base pair	• Amplification-free method • Bisulfite conversion free method • Low-sample input • Rapid throughput	• Less established • Few commercially available

BeadChip arrays are commonly used due to the high-throughput capability, relative economy per sample per CpG, reproducibility and the inclusion of regions of known function and disease significance. For example, the most recent version Illumina Human MethylationEPIC array covers over 850,000 CpG sites at a cost of approximately US$480 per sample [28]. The EPIC array incorporates a greater number of CpGs in the open sea, cytosine nucleotide guanine (CNG) sites and enhancers identified in FANTOM5 (functional annotation of the mammalian genome 5). The probes cover approximately 3% of CpG sites in the genome, but more than 70% of the RefSeq (NCBI reference sequence database) identified transcriptional start sites (TSS) 5′UTR and 3′UTR CpG sites [28]. Novel methods for DNA methylation detection using optical or electrochemical biosensing are being developed, alongside single cell technology. Selection of a DNA methylation measurement method should balance the practicalities of cost alongside the coverage and specificity offered.

## Analysing DNA methylation data

Genome-wide methylation studies are complex. Investigators will often seek to use pre-existing cohorts for experiments. This is not recommended as statistical methods cannot compensate for complete confounding variables, for example where all cases are smokers and all controls are non-smokers.

### Quality control

Data from DNA methylation measurements differ depending on the type of experiment. Array-based methylation can be expressed as either *β* values, the ratio of methylated probe intensity to overall probe intensity, or *M* values, the log_2_ of the ratio of methylated to unmethylated probes. *M* values are preferable for statistical analysis, whereas *β* values are suited to displaying data. In sequencing-based experiments, coverage, or tag count, can be used as a measure of methylation.

Batch variation is particularly an issue with highly sensitive methods, such as BeadChip arrays. Whilst in silico methods can be used to overcome batch variation [29], it is always preferable to control for batch variation through properly designed experiments and sample randomisation. Recommendations for sequencing coverage vary between methods, largely influenced by the number of nucleotides assayed. Guidelines for coverage have been developed by the NIH Roadmap Epigenomics project and the ENCODE project [30, 31]. The most common method of DNA methylation measurement, Illumina BeadChip arrays, requires specific QC prior to analysis. Illumina provides Genome Studio for processing DNA methylation data, although several free analysis pipelines are available. For enrichment-based high throughput sequencing assays, measuring the CpG enrichment within the captured DNA is a useful QC step. Cut-offs for individual CpGs (or regions) should be applied, as sufficient average coverage does not guarantee sufficient coverage of individual CpGs.

If publically available datasets or previous versions of arrays are to be used, careful QC should be carried out to curate the datasets to samples controlling for phenotypic differences and for plate variation. Illumina BeadChips may be compared due to the high level of similarity between different iterations, but plate variation is always an issue.

### DNA methylation analysis

The major approaches to analysing changes in DNA methylation are as individual CpGs (differentially methylated positions (DMPs)) and differentially methylated regions (DMRs). DMR can be impactful due to the close regulation of proximally located CpGs; however, interpreting DMR can be difficult if regions contain both hypo- and hyper-methylated positions, due to the function of specific regions (e.g. effector vs promoter regions). CpGs can be easily defined as increased or decreased due to the binomial distribution, although the increasing number of CpGs being analysed requires a large sample size to achieve statistical significance after multiple testing corrections, such as the Benjamini-Hochberg false discovery rate (FDR).

Whilst it is always preferable to control for measured covariates through experimental design, various in silico methods have been developed to identify DMPs whilst controlling for confounding variables. These include linear regression methods incorporating initial tests for confounding variables such as either principal component analysis (PCA) or multi-dimensional scaling (MDS). The recently developed OSCA method was specifically developed for methylation-association studies, employing a mixed linear model-based method to detect DMPs whilst fitting all other probes as random-effect components to account for the effects of confounders [32]. This approach has been shown to control for observed and unobserved confounders better than standard approaches, retaining statistical power whilst avoiding inflation of association findings, and is an important advance in the field.

DNA methylation is co-regulated alongside other epigenetic data, such as gene expression and chromatin accessibility. Therefore, examining DNA methylation within the context of other ‘omics data, such as disease variants to identify methylation quantitative trait loci (meQTL), enables greater functional understanding of DNA methylation changes. These datasets can be used to create a comprehensive map of genetic, transcriptional and epigenetic data, and the relationships between each other. Network analysis can then be applied to search for pathways enriched for disease-related changes.

## DNA methylation studies in AS, IBD and psoriasis

The current state of DNA methylation studies in AS, IBD and psoriasis provides information on previously identified shared pathways and avenues for future research. As these diseases have been found through genotypic studies to have pleiotropic genes (the same gene with different effects on function), it is more useful to discuss each disease individually and then address overlapping pathways. The key studies are summarised in Table 2.

**Table 2:** Seminal papers on DNA methylation in IBD, psoriasis and AS. Study design elements and key findings are outlined

Condition	Reference	Study design	Patients	Controls	Tissue	Measurement method	Key findings
Inflammatory bowel disease	Hasler [33]	MZ CC	20 UC discordant MZ twins 50 inflamed UC; 30 non-inflamed UC	50 HC 25 inflamed disease controls 30 non-inflamed disease controls	Intestinal mucosa tissue	Illumina Methylation 27 BeadChip Custom tiling array; pyrosequencing	Integrated DEG data, MeDIP-seq data and Illumina Methylation 450K data was used to identify 61 IBD-associated loci harbouring DMP in cis of a DEG. All were novel candidate risk loci for IBD including SPINK4, THY1 and CFI.
	McDermott [34]	CC	150 IBD patients; 24 paediatric IBD	40 HC; 22 paediatric HC	PBMC; colonic mucosa tissue	Illumina Methylation 450K BeadChip; pyrosequencing	3196 probes were significantly differentiated between CD patients and healthy controls, and 1418 probes between UC patients and controls. The most significant DMP for both groups was in the 5′UTR of TIFAB.
	Ventham [35]	CC	240 IBD patients	74 symptomatic HC 117 HC	Whole blood, CD4+ T cells, CD8+ T cells, CD14+ monocytes	Illumina Methylation 450K BeadChip	Samples clustered by cell type separately from PBMCs. The top DMP in PBMCs was driven by changes in CD14+ monocytes, not either T cell subset. Conversely, some significant DMP in cell subsets were not detectable in whole PBMC. DMP were significantly associated with known IBD-risk loci.
	Howell [36]	CC	66 paediatric IBD	30 paediatric HC	Bowel mucosal biopsies	Illumina Methylation 450K or EPIC BeadChip; pyrosequencing	Studied DNA methylation, gene expression and gut microbiota from a single cohort at multiple intestinal sites. Site-specific signatures were observed for DNA methylation and gene expression in the ascending colon and sigmoid colon compared to the terminal ileum.
	Somineni [37]	CC	164 paediatric CD	74 paediatric HC	Peripheral blood	Illumina MethylationEPIC BeadChip	Previous findings were replicated including *VMP1*, *RPS6KA2*, *ITGB2*, and *TXK*, alongside known therapeutic targets (*TNF*, *JAK3*, *IL12B*, *IL23A*, and *IL1R1*). 194 DMP had a strong evidence of genetic effect. The Crohn’s methylation signature was a powerful predictor of disease (AUC = 0.91), but had no prognostic utility.
Psoriasis	Gervin [38]	MZ	27 MZ pairs discordant for psoriasis	27 HC	CD4+ cell and CD8+ cell from PBMCs	Illumina Methylation 27 BeadChip	DNA methylation highly correlated between monozygotic twins in CD4+ T cells and CD8+ T cells. No differences were identified between individual CpG sites or overall methylation per gene. When combined with gene expression data, cell-specific differences were identified, including IL13, ALOX5AP, PTHLH and TNFSF11.
	Zhou (1) [39]	CC	114 psoriasis patients	62 HC	Skin biopsies PP and PN	Illumina Methylation 450K BeadChip; Sequenom Epityper system	129 SNP-CpG pairs achieved statistical significance between psoriatic and healthy control peripheral blood, constituting 28 unique meQTLs and 34 unique CpGs. 11 SNP-CpG pairs passed CIT constituting 3 unique CpG sites within C1orf106, DMBX1 and SIK3.
	Zhou (2) [40]	CC	114 Psoriasis patients (41 PP+PN)	62 HC	skin biopsies; Previous methylation data	Illumina Methylation 450K BeadChip	1514 DMP were identified between PP and PN, only 426 DMP were identified between PP and PN, and none between PN and NN. During replication, 9 sites reached significance (*CYP2S1*, *ECE1*, *EIF2C2*, *MAN1C1*, *DLGAP4*, *S100A9*, *S100A7A* and *S100A5*), but none replicated in PBMCs.
	Pollock [41]	CC	23 psoriasis patients without PsA; 13 PsA individuals	18 HC	Sperm	Illumina Infinium Human Methylation 450K BeadChip	2467 DMP identified between PsA and healthy controls, compared to 574 DMP between psoriasis and controls. DMR were enriched for the MHC complex. *IL22* was associated with joint and skin involvement and was the only site to have correlated methylation levels between sperm and blood.
Ankylosing spondylitis	Aslani [42]	CC	40 AS	40 HC	PBMC	MSP	DNMT1 promoter was hypermethylated in cases compared to controls. No significant difference between HLA-B*27+ and HLA-B*27- patients, or disease activity scores.
	Karami [43]	CC	50 AS	50 HC	PBMC	MSP	Cases had decreased *BCL11B* expression associated with increased methylation in the promoter region.
	Hao [44]	CC	10 AS	10 HC	PBMC	Illumina Methylation 450K BeadChip	1915 CpGs were identified as differentially methylated between cases and controls. The most significant was HLA-DQB1, previously associated with AS radiographic severity and age of onset. Increased DNA methylation associated with decreased HLA-DQB1 expression.

### Ankylosing spondylitis

The first DNA methylation study in AS was published in 2014, and there has been a scarcity of studies since that time. Only a single multigene study has been published with a small cohort of 5 individuals with grade 4 bilateral sacroiliitis, complete fusion of both sacroiliac joints and 5 age- and sex-matched controls [44]. 1915 DMP were identified, the most significant of which was *HLA-DQB1*, a MHC class II responsible for exogenous peptide display, which had previously been associated with AS radiographic severity and age of onset, but not within Han Chinese individuals. Unfortunately, this study failed to account for HLA-B*27 status, the strongest AS-associated genetic loci, which is in strong LD with *HLA-DQB1* [45]. Therefore, these findings require further validation.

The single-gene studies have been a mix of genetically associated and broadly inflammatory-related genes: the suppression of cytokine signalling 1 (SOCS1*), *DNMT1* [42], *B-cell chronic lymphocytic leukaemia/lymphoma 11B* (*BCL11B*) [43], *IFN regulatory factor 8 (IRF8)* [46] and IL12B* [47] (*genetically associated with AS). The study on *SOCS1* acknowledged that the differences observed in *SOCS1* in cell-free DNA was likely due to inflammation-driven cellular apoptosis in AS patients. The study of *IL12B* tested the performance of methylation as a biomarker for AS. The area under the curve of the ROC analysis was statistically significant but not of sufficient magnitude to be clinically useful (AUC = 0.65), and no validation study was done. The methylation differences were restricted to HLA-B27 positive cases, suggesting either that the methylation is affected by HLA-B27 status or that the sample size of HLA-B27-negative cases (*n* = 16) was too small. As the HLA-B27 status of the controls was unknown, it is unclear which explanation applies [47]. None of the studies investigated whether the AS-associated SNPs at these loci operated through effects on methylation.

Whilst many of these studies attempted to connect these changes with HLA-B*27 status, none performed HLA-B*27 typing in healthy controls, who generally have a significantly lower prevalence of HLA-B*27. All focused on promoter regions and identified inverse correlations with mRNA levels for the affected gene. Thus, for the majority of the 116 known AS-associated variants, there is no robust information about methylation effects. AS is known to be associated with *DNMT3A*, *DNMT3B* and *DNMT3L* [4], indicating that it is likely that methylation variation is important in AS pathogenesis. Overall, only a sparse investigation of DNA methylation has been performed in AS to date, and those studies have suffered from failures to collect appropriate information at study onset (such as HLA-B*27 status) or to use appropriate sample types.

### Inflammatory bowel disease

DNA methylation research in IBD is the most robust of the three diseases, with over 30 papers published in the last decade. There is strong indirect evidence that methylation effects are likely to play a significant role in IBD pathogenesis, with 33% of the heritability of ulcerative colitis, and 30.5% of the heritability of Crohn’s disease, being associated with SNPs affecting methylation levels (mQTL) [48]. IBD studies have had a strong focus on DNA methylation as a biomarker for progression to colorectal cancer [49–51]. In this review, we will be focusing on those studies that investigate methylation involvement in the pathogenesis of IBD itself. The differences between the IBD subsets, ulcerative colitis and Crohn’s disease have been heavily studied. Consistent with the substantial genetic overlap between these subsets, multigene DNA methylation studies ascertained overlap in the differentially methylated genes in both subsets [34, 35, 52–58].

Multigene DNA methylation studies in IBD have been highly variable in terms of the genes observed. Similar pathways have been identified including those related to tissue/skeletal morphogenesis (e.g. *PRICKLE1*, *SOX11*, *TGFBR3*, *NKX2-3*) [54, 56, 58, 59], immune pathways such as the Wnt/NF-κβ (e.g. *PITX2*, *RARB*, *ROR1*, *FOXA2*) [58, 60], IL-23 pathways (e.g. *STAT3*, *BCL3*, *OSM*, *TLR4*) [53, 61] and inflammation-associated genes (e.g. *ITG1B2*, *SAA1*, *IFITM1*, *ITGB2*) [54, 55, 58, 62, 63].

Methylation studies in IBD are challenged by the accessibility of tissues, variability of disease treatment and obtainment of appropriate controls. Medication usage in IBD treatment varies to a greater extent than those used in psoriasis and AS, complicating analysis and potentially explaining the failure for most studies to consider it as a covariate. Uninflamed and healthy control biopsies are often obtained from different gastrointestinal regions to the inflamed samples. This introduces variation based on the location of samples which can confound disease associations with location differences. Howell et al. specifically examined this issue and identified site-specific signatures for DNA methylation and gene expression between the ascending colon and sigmoid colon compared to the terminal ileum [36]. Signatures in each region were also found to be disease subset specific, which agrees with the differential distribution of each subset within the gastrointestinal system. This variability is likely also linked to the different cell types within each region. Ventham et al. examined both whole blood and isolated immune cell subsets (CD4+ T cells, CD8+ T cells and CD14+ monocytes) [35]. Gene expression and DNA methylation were highly clustered by cell type in the principal component analysis (PCA), and all individual cell types clustered separately from whole blood. The signals identified in whole blood were often diluted cell-specific signals, as seen with *RPS6KA2*, the top DMP in whole blood, which was only significantly differentially methylation in CD14+ monocytes. Conversely, *HDAC4* which was also significantly differentially methylated in CD14+ monocytes was not significantly different in whole blood. Ventham et al. further demonstrated that IBD-associated DMP co-localised with known IBD-associated GWAS loci, significantly more than randomly generated bins with similar probe density [35]. These IBD-associated DMPs were used to identify 326 cis meQTLs, with two SNPs associated with *VMP1* both in linkage disequilibrium with a known IBD-susceptibility allele (rs1292053). This study used causal interference testing (CIT) to investigate if DNA methylation was a mediator between *VMP1* genotype and phenotype or if this was independent or consequential to genotype [35, 64]. The authors cited insufficient sample size as a likely reason for the inability to prove the relationship, an issue echoed by many papers in all three diseases. As this is the largest DNA methylation study in IBD, psoriasis or AS, it indicates the need to reassess the current sample sizes considered for DNA methylation.

Somineni et al. recently reported on methylation differences in peripheral blood mononuclear cells in 164 treatment-naive paediatric IBD cases and 74 non-IBD controls [37]. They found 1189 differentially methylated sites (false discovery rate < 0.05) and replicated Ventham’s previous findings at *VMP1*, *SBNO2*, *RPS6KA2*, *ITGB2* and *TXK.* Of these, 194 showed genetic effects on the change in methylation, and using Mendelian randomisation approaches, they found evidence to suggest that three of these, two involving the gene *GPR31* and one involving *RNASET2*, showed evidence of a causal relationship with Crohn’s disease, replicating a prior finding at *RNASET2* [65]. Their Crohn’s disease methylation signature overlapped strongly with previously reported signatures associated with the chronic low-grade inflammatory disease as assessed by serum CRP measured in subjects with a broad range of diseases including cardiovascular disease and diabetes. Finally, they demonstrated that the methylation signature was a powerful discriminator between cases and controls (AUC = 0.91), but had no utility in predicting prognosis. Howell et al. similarly found that methylation signatures had the high discriminatory capacity in paediatric IBD, with sigmoid colon methylation having AUC = 0.94 [36]. Whether this signature is as predictive in subjects with suppressed disease, or in comparison with other forms of colitis, and the extent of overlap in the methylation signatures these two publications employed, awaits further study.

The link between IBD-associated genotypes and DNA methylation has also been investigated in several single-gene studies. The ulcerative colitis severity region-associated rs1861494 T allele in *IFNG* was associated with this *IFNG* promoter methylation loss, and more severe disease outcomes in both ulcerative colitis and Crohn’s disease [66]. *NKX2-3* (rs1190140), *IL-17A* (IVS1+18) and *STAT4* (rs7574865) all cause loss or gain of a CpG site [61, 67, 68]. In *STAT4* and *IL17A* the T risk alleles result in loss of CpG sites and associated increased expression of the cytokine in both colonic tissue and PBMCs [68]. These studies have provided insight into the mechanisms through which these SNPs can affect function; however, they are limited in their coverage and only encompass loci associated with coding regions.

### Psoriasis

Fewer methylation studies have been performed in psoriasis than IBD. Psoriasis has the benefit of a relatively accessible and easily defined sample types, skin and T cells (known to play a primary role in psoriasis pathogenesis) [38, 69–73]. Additionally, most of the studies in psoriasis have focused on multiple genes, likely enabled by the smaller number of genetic loci associated with psoriasis (~ 60 loci) [3]. This has resulted in a focus on integrated datasets, such as gene expression and genotyping. As expected, the differentially methylated genes identified in the skin are mostly associated with epidermal and keratinocyte differentiation, and cellular regeneration [39, 74–76]. Four skin biopsy-based studies identified members of the *S100A* family, part of the epidermal differentiation complex, as top differentially methylated hits [39, 40, 75–78]. Many studies have failed to identify differences in skin biopsies in affected non-inflamed skin compared to healthy controls [39, 75, 76]. It is therefore worth considering if the skin-related changes in psoriasis are not causative, but rather responsive to psoriasis pathogenesis. Only Zhou et al. examined both skin punch biopsies and PBMCs, and none of the psoriasis-associated DMP replicated between the sample types [39].

Differentially methylated genes in immune cell types have differed from those associated in skin; however, the pathways identified in both samples encompass cellular adhesion and intracellular signalling pathways and inflammatory pathways (such as the IL-23 and STAT/JAK signalling pathways which are consistent with psoriasis-associated genetic variants (e.g. *IL13*, *ALOX5AP*, *PTHLH* and *TNFSF11*) [38, 70, 75, 76]). This was observed in Zhou et al. (2017) where 3 unique CpG sites were identified with 11 associated SNP-CpG pairs within *C1orf106*, *DMBX1* and *SIK3* [39, 40, 64]. C1orf106 regulates adherence junction stability, although the key gene at this locus remains unclear [79]. SIK3 is associated with an mTOR signalling cascade involved in skeletogenesis and is an upstream regulator of HDAC. DMBX1 is involved in cell cycle regulation. Unfortunately, this was the only study to incorporate both genetic and DNA methylation analysis, and thus, for most psoriasis-associated loci, there is no information as to whether genetic variants operate through methylation to influence disease. The few single-gene studies have identified differential methylation in the promoter regions *of p15*, *p16*, *p21*, *ID4*, *IFNG* and *HLA-C* [69, 72, 77, 78, 80]. All had associated changes in gene expression. PASI score is associated with increased p16 and HLA-C promoter methylation and decreased *HLA-DRB1* promoter methylation [77, 78, 81]. These findings suggest that more comprehensive studies of methylation in psoriasis are likely to be productive.

A novel study examining methylation variation in the sperm in psoriasis (PsC), psoriatic arthritis (PsA) and healthy controls aimed to determine if the parent of origin heritability effects in these diseases were due to methylation effects [41]. Studying the sperm rather than peripheral blood or skin also obviated the issue of cellular heterogeneity. At a false discovery rate < 0.05, 574 differentially methylated sites were observed comparing PsC patients and controls, 2467 between PsA patients vs. controls and 342 between PsA and PsC patients. The major histocompatibility complex was enriched for these sites, and there were several strong biological candidates for involvement in these diseases amongst the sites. Whilst several CpG were examined in the blood, only IL22 had a direct correlation between methylation levels in both tissues. Whether the changes observed in the semen correlate with the changes in cell types likely to be involved in psoriasis will require further study.

## Limitations of previous studies

Overall, the studies examining DNA methylation in IBD, psoriasis and AS have suffered from similar issues. Cohort selection has often been driven by practical considerations; however, there has also been an insufficient recording of these factors for in silico analysis or adjustment. Smoking is a known risk factor for these diseases and affects disease severity and treatment response, yet only 5 IBD studies even recorded smoking status. An overarching theme is the insufficient sample sizes used, cited by most papers as interfering with identification of disease-associated signals. This is linked to the ongoing issue of sample type used (tissue or circulating cells). A question which is yet to be addressed is the most appropriate sample type in each disease. The frequency with which these issues are discussed as a potential barrier to analysis indicates that these issues are pervasive in the study design for these diseases.

## Best practices

This review of studies in these three related diseases highlights some high-quality publications and also that there are a high number of isolated findings which have either not replicated, or no replication study has been performed of them. It is clear from this review that for the field to make meaningful contributions to health research, some simple, well-defined best practice guidelines are needed.

### Study focus

DNA methylation studies generate a large amount of data and are affected by numerous factors. Therefore, it is important to clearly define an achievable question or goal for this information from the outset. This will also enable the collection of additional functional information, such as gene expression or genotype, where possible.

### Cohort selection

Reproducibility and statistical power are best when variations in factors that affect DNA methylation exogenous to the hypothesis being tested are controlled for. The factors outlined above should be considered when planning what criteria will be used for cohort selection and analysis. Age and sex should be matched between case and control cohorts; however, other factors including smoking and medication should be controlled where possible or recorded when not. Medication can be controlled through selecting for treatment naïve individuals, using paired samples pre-and post-treatment, matched case studies or controlling for medication use in silico (where appropriate).

Although these factors can be adjusted for in silico each factor that has to be adjusted for reduces the power of the study to identify disease-relevant signals. Therefore, proper cohort selection is always preferable to in silico methods.

### Sample type

Sample types are often selected due to practical considerations such as accessibility, invasiveness and expense. This has led to a broad use of circulating immune cells. However, a key assumption is that the profiled tissue is the most disease relevant. The primary site of the disease remains contended in these diseases and no robust comparison of circulating and tissue resident cells has been performed, leaving the utility of circulating cells as substitutes for primary disease site largely unknown. Whilst these circulating cells may be relevant to disease, they may not encompass tissue-specific methylation changes relevant to disease. It is therefore imperative to examine cell types in isolation, to enable comparisons between tissue resident and circulating cells, and to control for cell-specific DNA methylation patterns.

### Sample size

**Fig. 2:**
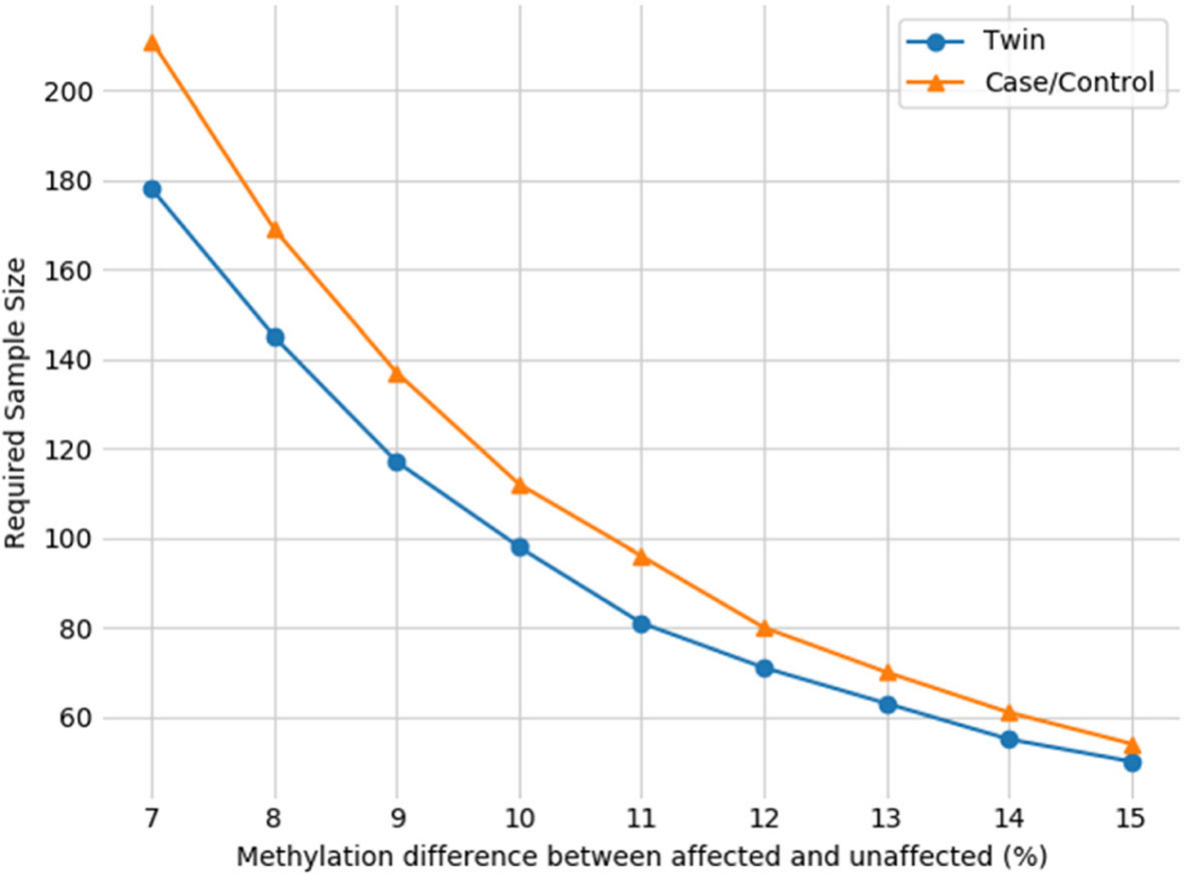
Sample size requirements for genome-wide significance. Estimated sample sizes, expressed as the number of pairs, either twin pair or case-control, required to reach 80% power in twin and case/control designs using a genome-wide significance threshold of 1 × 10^−6^. Data taken from Tsai and Bell 2015

Due to a large number of statistical tests now being carried out in multi-gene studies, stringent levels of genome-wide significance should be sought. Various cut-offs have been suggested in the literature: 1 × 10^−6^ [82], 1 × 10^−7^ [83] and 5 × 10^−8^ [84]. A 10% change in DNA methylation at a single CpG would require 21 paired case controls, but the same change at genome-wide significance (1 × 10^−6^) requires over 100 case-control pairs (Fig. 2). Multiple testing adjustments should be included in these calculations. Sample size requirements should be determined prior to the study outset using either pilot studies or similar cohorts to determine effect size (methylation different between groups).

### Method selection

Although over a third of studies in IBD, psoriasis and AS used MSP, it is recommended that this method only be selected where candidate genes have already been identified and the method is being used to investigate the relationship between genotype or gene expression with methylation of that gene. Otherwise, a multigene approach is always preferable. Each method has individual advantages and disadvantages, as outlined in Table 1. It is important to consider the limitations of each method prior to selection. This should then influence the analysis and quality control steps that will be used. If data is to be integrated with another study, care should be taken to select similar cohorts and methods for measurements.  Variation due to differences in the factors discussed above should be tested prior to analysis. It may be beneficial to treat these as a validation cohort rather than to integrate such data.

### Integration with ‘omics data

It is informative to examine DNA methylation in the context of genomic or transcriptomic data as this provides context and functional information. It also provides a method to prioritise DMP. Currently, there has been limited integration of genotype with DNA methylation data. This has made it difficult to identify the relationship between the genetic basis of these diseases and changes at a DNA methylation level. It is also suggested that the integration of microbiota and cytokine data be considered, due to the strong link between gut microbiota changes and all three diseases.

### Replication/validation

Technical verification is recommended prior to undertaking biological validation, as it is generally less time consuming and expensive than biological validation. PCR-based techniques are commonly used for technical validation of BeadChip arrays. It is noteworthy that only twin studies or juvenile cohorts carried out biological validation [33, 52, 54]. This is particularly relevant in DNA methylation due to high levels of inter-individual variation and tissue-specific DNA methylation patterns.

## Conclusion

Despite evidence in twin studies that epigenetics is a major mechanism through which immune-mediated disease-associated variants affect function, the role of DNA methylation, the most well-characterised epigenetic mechanism, in immune-mediated diseases remains largely unknown. DNA methylation remains an under-investigated biological mechanism in AS, IBD and psoriasis. Similar pathways have been identified as differentially methylated in each of these diseases, and most have been previously identified through genetic association studies. However, studies to date have been limited by study design issues, including poor cohort selection, improper controls and insufficient statistical power.

Future studies should incorporate the factors outlined in this review and take steps prior to study outset to perform sample size calculations, cohort selection criteria, and the selection of appropriate measurement techniques and analysis pipelines that are based around those techniques. These should be followed by biological and technical validation to ensure that results are held to a robust standard. The use of consistent and appropriate experimental design will enable the identification of disease-relevant changes in DNA methylation and provide functional information to inform rational treatment design in these immune-mediated diseases.

## Data Availability

Not applicable

## Abbreviations

5hmC: 5-Hydroxymethylcytosine
5mC: 5-Methylcytosine
AS: Ankylosing spondylitis
BMI: Body mass index
CC: Case-control study
CD: Crohn’s disease
CIT: Causal interference testing
CNG: Cytosine nucleotide guanine
COBRA: Combined bisulfite restriction analysis
CpG: Cytosine phosphate guanine-paired bases
DMP: Differentially methylated position
DMR: Differentially methylated region
DNMT: DNA methyltransferase
FANTOM5: Functional annotation of the mammalian genome 5
FDR: False discovery rate
FRET: Fluorescence resonance emission transfer
GWAS: Genome-Wide Association Study
HC: Healthy control(s)
HELP-tagging: HpaII tiny fragment enrichment by ligation-mediated PCR
HLA: Human leukocyte antigen
IBD: Inflammatory bowel disease
LINE: Long interspersed nuclear element
MDS: Multi-dimensional scaling
MeDIP-seq: Methylated DNA immunoprecipitation sequencing
meQTL: Methylation quantitative trait loci
MIGS: Methyl-CpG-binding domain-isolated genome sequencing
MSAM: Methylation-specific amplification microarray
MSP: Methylation-specific PCR
MZ: Monozygotic twin study
NN: Normal non-psoriatic samples
NSAIDs: Non-steroidal anti-inflammatory drugs
PBMC: Peripheral blood mononuclear cells
PCA: Principal component analysis
PN: Psoriatic uninvolved samples
PP: Psoriatic involved samples
QC: Quality control
RefSeq: NCBI reference Sequence database
RRBS: Reduced representation bisulfite sequencing
scNMTseq: Single-cell nucleosome, methylation and transcription sequencing
SPR: Surface plasmon resonance
TDG: Thymine DNA glycosylase
TET: Ten-eleven translocation
TSS: Transcriptional start site
UC: Ulcerative colitis
WGBS: Whole genome bisulfite sequencing
